# Plant mass variations of *Leymus chinensis* (Poaceae) and their relationships with environmental factors on a large‐scale gradient, northeastern China

**DOI:** 10.1002/ece3.11215

**Published:** 2024-05-15

**Authors:** Yuebin Zheng, Jing Xue, Yixia Lv, Chaoxue Zhang, Renzhong Wang

**Affiliations:** ^1^ State Key Laboratory of Vegetation and Environmental Change, Institute of Botany the Chinese Academy of Sciences Beijng China; ^2^ University of Chinese Academy of Sciences Beijng China

**Keywords:** climate effect, large‐scale drought gradient, *Leymus chinensis*, plant mass, soil nutrient and microbial diversity

## Abstract

Body size (or mass) variations and their relationships with environmental variability have been well documented for many species at the local scale, while the effects of climate, combined with soil nutrients, on plant mass in large‐scale gradient remain unclear. Herein, detailed surveys were conducted to investigate plant mass (PM, aboveground mass per plant) variations of *Leymus chinensis* and their relationship with environmental factors (e.g., climate, soil nutrient, and microbial diversity) at 18 wild sites along a large‐scale gradient from 114 to 124° E in northeastern China. Based on site‐by‐site analyses, the plant mass of the species varied significantly from east to west along the gradient. It initially increased, peaking at middle sites, and then dropped with the increase of drought in both dry and rainy seasons. Plant mass at the eastern end was almost equal to that at the western end and was equivalent to 1/2 and 1/3 of middle sites. The average plant mass in the rainy season was about 50% greater than that in the dry season (*F*_1,1078_ = 489.80, *p* < .001). The effects of environmental variables on plant mass differed in dry and rainy seasons. Mean annual temperature and temperature seasonality were the critical restrictions of plant mass in the dry season, while temperature and precipitation seasonality and soil resources (total C, Mn, Zn) had significant impacts in the rainy season (*p* < .05). In general, plant mass had not dropped linearly with the increase of drought along large‐scale gradient, suggesting that precipitation decrease was not the critical restriction regulating the growth and settlement of the species.

## Graphical abstract

The specific aims were to examine plant mass variation of *Leymus chinensis* (Poaceae) and resolve its main limiting factor, address the regulation mechanism between plant mass and population density at steep moisture gradient, and predict the influence of climate change on plant distribution in the future.

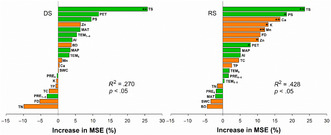

## 1 INTRODUCTION

Large‐scale environmental variability (e.g., drought and climate warming) exerts a key factor limiting plant distribution both directly through influences on plant growth, reproduction, and establishment, and indirectly through effects on climatically related disturbances, such as fire and insect outbreaks (Callaway et al., 1994; Villalba & Veblen, 1998). Plant adaptation strategies (e.g., density and PM regulation) often differ and are complex due to various environmental factors (e.g., precipitation, temperature, and soil factors) at large scales. Understanding the relationships of plant strategies to large‐scale spatial environment variations can yield important information regarding to the effects of climate changes on species shift, ecosystem structures, and functions in the future (Chaves et al., 2003; Wang & Gao, 2003; Wang & Ma, 2016).

Most studies on the effects of environmental variability have focused on the linkage between climatic changes, plant populations (Wang & Gao, 2003; Yuan et al., 2016), and morphological traits (Cardou et al., 2022; Griffin‐Nolan et al., 2023; Hounkpevi et al., 2020). Wang and Gao (2003) found that *Leymus chinensis* adjusts to decreasing mean annual precipitation (MAP)/increasing aridity by altering shoot density and biomass allocation along large‐scale drought gradients (Wang & Gao, 2003). Similarly, demographical, morphological, and physiological phenotypes of 18 *L. chinensis* populations (in wild sites) exhibited significant divergence along large‐scale gradients, but these divergent variations narrowed significantly at the transplantation, indicating that divergent selection/climate variable (mainly MAP) was the main factor in determining the phenotypic divergence of the species along the large‐scale gradient (Yuan et al., 2016).

Recent research has explored the interactions of plant–soil nutrient and microbial diversity and their effects on PM and ecosystem function and stability (Gao et al., 2020; Wang et al., 2022). Root vertical distributions and biomass (root mass) for two *Artemisia* species positively correlated with soil water, total soil nitrogen, and carbon contents, suggesting that both soil moisture and poor soil nutrients were the limiting resources for the growth and settlement of the two species in dry regions at a local scale (Gao et al., 2020). At large‐scale edaphic and precipitation gradient, plant biomass and diversity were positively correlated with soil fungi diversity, particularly for predicted arbuscular mycorrhizal fungi and saprotrophic fungi, and they were positively related to soil nutrients and texture (Wang et al., 2022). However, the interactions of PM for cosmopolitan plant species with zonal changes in soil nutrient and microbial diversity were not clear.

Although few studies have looked at the relationships of body size with population density, abundance, as well as climate change, or geography (Brown et al., 1999; Poulin, 1999; Schmid et al., 2000), there is less investigation about the influences of environmental variations on PM of nature species along large spatial scale (>1000 km). This knowledge is essential for understanding the regulatory mechanisms of plant distribution, such as the balance between PM/growth and density during drought stress, and predicting the effects of climate change on plant growth and distribution in the future (Lande, 2009).

*Leymus chinensis* (Trin.) Tzvel. (Poaceae), a perennial high‐quality forage, is widely distributed at the eastern end of the Eurasian steppe zone, mainly from the moist part of the western Northeast Plain to the dry part of the eastern Mongolian Plateau, China (Wang & Gao, 2003). The xerophytic traits (e.g., thick rhizome systems and high plasticity in leaf traits) enable the species to successfully tolerate or resist drought when soil moisture is less than 4% in dry seasons on the western desert steppes (Wang & Gao, 2003; Yuan et al., 2016). Previous research has demonstrated that drought or decrease in precipitation was the main restriction of population density, biomass, and biomass allocation of the species along the gradient (Chen & Wang, 2009; Wang & Gao, 2003). Wide distribution and fast growth make this species an ideal plant for studying the change patterns of PM/growth at a large‐scale gradient (spatial scale), but also for predicting the influence of climate changes on PM/growth in the future (temporal scale). We hypothesize that PM at the gradient was confined to mean annual precipitation (MAP), precipitation from January to May (PRE_1–5_), and June to August (PRE_6–8_). The specific aims were to examine PM variation of the species and resolve its main limiting factor, address the regulation mechanism between PM and population density at steep moisture gradient, and predict the influence of climate change on plant distribution in the future.

## 2 MATERIALS AND METHODS

### 2.1 Study sites

Experiments were conducted on native *L. chinensis* grasslands in 2021. These grasslands are distributed on the Northeast plain and Inner Mongolian plateau, along a large‐scale longitude gradient, ranging from 43°16′ to 44°36′ N and from 114°51′ to 124°14′ E, about 1000 km from the west to the east. Due to the steep increase of precipitation from the west to the east, vegetation varies gradually from desert and typical steppes in the west to agricultural fields and meadow grasslands in the east of the gradient (Wang & Gao, 2003; Yuan et al., 2016). Most of the *L. chinensis* grasslands have dark brown soils, saline meadow soils, and chernozems in the east, while those of desert and typical steppes have chernozem and chestnut soils in the west (Wang & Gao, 2003). The elevation along the gradient drops from 1321 m (asl.) in the west to 148 m (asl.) in the east (Table 1).

**TABLE 1:** Sample locations, vegetation types, soil type and climate variables.

Site	No.	Latitude N	Longitude E	Vegetation type	Soil type	Elevation (m)	MAP (mm)	MAT (°C)	Aridity index
Wulantuga	01	44°29′	124°14′	Meadow	Dark meadow	177	471	5.40	0.38
Chaganhua	02	44°30′	124°06′	Meadow	Dark meadow	163	462	5.59	0.36
Wulanaodu	03	44°36′	123°48′	Meadow	Dark meadow	153	446	5.84	0.34
Beizheng	04	44°28′	123°30′	Meadow	Dark meadow	150	437	6.15	0.33
Taipingchuan	05	44°21′	123°14′	Meadow	Dark meadow	148	472	6.34	0.35
Baolongshan	06	43°58′	122°44′	Meadow	Chernozem	157	439	6.77	0.31
Liaohe	07	43°45′	122°23′	Meadow	Chernozem	170	431	6.78	0.30
Molimiao	08	43°34′	121°55′	Meadow	Chernozem	179	443	6.98	0.31
Shaogen	09	43°38′	120°47′	Meadow	Chernozem	278	407	7.07	0.27
Tianshaneast	10	43°50′	120°15′	Meadow	Chernozem	424	391	7.10	0.26
Tianshanwest	11	43°50′	119°59′	Meadow	Chernozem	520	390	6.66	0.26
Bayantala	12	43°42′	119°03′	Steppe	Chernozem	789	389	5.01	0.28
Xinchengzi	13	43°20′	118°12′	Steppe	Chernozem	776	366	5.70	0.27
Dalainuori	14	43°16′	117°10′	Steppe	Chernozem	1321	362	0.73	0.32
Baiyinxile	15	43°31′	116°41′	Steppe	Chernozem	1319	330	0.76	0.28
Maodeng	16	44°08′	116°21′	Steppe	Chernozem	1143	278	1.76	0.22
Baoligen	17	43°55′	115°44′	Desert grassland	Chestnut	1094	257	1.87	0.20
Bieligutai	18	43°59′	114°51′	Desert grassland	Chestnut	1128	228	1.56	0.17

Abbreviations: MAP, mean annual precipitation; MAT, mean annual temperature (hereafter for tables and figures).

### 2.2 Climate

The main determinants of the climate in the regions are the Mongolian anticyclone and the moist Pacific air mass. In winter, the area is dominated by an intense Mongolian anticyclone (Wang & Ripley, 1997). The steep pressure gradient between this high and the Aleutian low‐pressure system produces a strong westerly flow of cold, dry continental air over the area. As the anticyclone breaks down in spring, the region comes increasingly under the influence of moist Pacific air masses, reaching a climax in the summer monsoon, which lasts for 2 months. As the summer draws to an end, the low‐pressure area over the Indo‐Pakistan subcontinent disappears with the development of the Mongolian anticyclone (Domros & Peng, 1988). The mean annual temperature (MAT) ranges from 0.7 to 7.1°C, varying from −21°C in January to 23°C in July. The mean annual precipitation (MAP) varies from 228 to 472 mm, with less than 30% rainfall occurs from January to May (dry season, DS), while 70% falls from June to August (rainy season, RS), and aridity index ranges from 0.17 to 0.38 along the gradient from west to east (Table 1). A more detailed description of the climate in the region may be found in Wang and Gao (2003) and Zhang et al. (1997).

### 2.3 Survey design

Eighteen sites were selected, approximately for every longitude degree, for plant and soil sampling along the gradient. At least 2 ha of native *L. chinensis* grassland, dominated by large patches of *L. chinensis* consociations, was selected for sampling at each site. Each site was of uniform soil type, with an even distribution of *L. chinensis*. For each site, three plots (50 × 50 m each), about 20 m apart, were established and 10 large patches were selected for plant and soil sampling in each plot. In order to explore the impacts of climate variabilities on PM in dry and rainy seasons, plant and soil samples were taken twice in early May (DS) and late August 2021 (RS), respectively. The sites selected for the study have not been grazed, ploughed, fertilized, or burned for at least 10 years prior to 2021, but transient floods may occur in the eastern meadow grasslands.

### 2.4 Methods

One growing well (stem and leave intact and free of diseases) shoot was selected randomly and cut along the ground in each large patch, all plant samples in each plot were placed in perforated paper bags, and oven‐dried at 80°C to constant weight before weighing. In each plot, soil cores were collected at a depth of 20 cm with a cylindrical soil sampler (5 cm diameter) and immediately processed using a 2 mm sieve to remove debris and gravel, then soil samples were placed into two sterile plastic bags for microbial community and soil property measurements, respectively. Samples for soil microbial community analysis were stored in a refrigerator at −20°C, and the other for measuring soil properties were air‐dried and stored.

#### 2.4.1 Soil property measurements

Soil water content (SWC) was determined by using a drying method where each soil sample was measured for fresh weight, dried at 105°C for 48 h, then weighed. SWC was calculated using the equation:

$$\mathrm{SWC}=\left(\text{Fresh weight}-\mathrm{Dry}\;\text{weight}\right)/\mathrm{Dry}\;\text{weight}\times 100\%.$$

Elemental analyzer (Vario EL Cube CHNOS Elemental Analyzer, Elementar Analysensysteme GmbH, Germany) was used to measure soil total carbon (TC) and total nitrogen (TN). Soil total phosphorus (TP), manganese (Mn), zinc (Zn), calcium (Ca), and potassium (K) contents were measured by X‐ray fluorescence spectrometer (Panalytical AXIOS mAX, Malvern Panalytical Ltd, The Netherlands).

#### 2.4.2 Soil microbial community analysis

Deoxyribonucleic acid (DNA) was extracted from 0.5 g soil sample using a Power Soil DNA Isolation Kit (MoBio Laboratories, Carlsbad, CA) following the manufacturer's instructions. Bacterial 16S ribosomal RNA (16S) genes and fungal internal transcribed spacer (ITS) regions were amplified via polymerase chain reaction (PCR) using the primer pairs 338F/806R and ITS1F/ITS2 combined with adapter sequences and barcode sequences, respectively. The PCR products were purified using a QIAquick gel extraction kit (QIAGEN, Germany), and sequenced on an Illumina MiSeq 300 PE platform (Illumina, San Diego, CA, USA).

### 2.5 Data analysis

Linear regression analyses were performed to evaluate the relationship of PM with precipitation and average temperature for different time terms (e.g., MAP, MAT, TS, precipitation from January to May), soil nutrients (e.g., SWC, TC, TN), and microbial diversity (e.g., bacterial diversity (BD) and fungal diversity (FD)). In order to reduce the likelihood of type I error in multiple correlations, the *p* values of significant (*p* < .05) and non‐significant (*p* > .05) correlations were adjusted using Bonferroni correction (Wang et al., 2022). One‐way analysis of variance (ANOVA) followed by least significant difference (LSD) multiple comparisons was used to detect the differences in plant mass between sites and different seasons. To assess the relative importance of environmental variables (climate, soil nutrients, and soil microbial diversity) in driving PM, random forest analysis (a software for estimating of what variables are important in the classification) by the “randomForest” function (ntree = 5000) in the random Forest package (Liaw & Wiener, 2002) was performed. All statistics for PM and environment factors were analyzed using R software (v.3.5.1).

Climatic parameters from the 18 sites, including MAT, MAP, precipitation seasonality (PS), and temperature seasonality (TS), were extracted from the WorldClim global climate database (Fick & Hijmans, 2017) using the geographic coordinates. The potential evapotranspiration (PET) data were obtained from CGIAR‐CSI (Trabucco & Zomer, 2018). Then the aridity index (AI) was calculated using the ratio of MAP to PET. In addition, the climate data of 2021, including temperature and precipitation from January to May (TEM_1–5_, PRE_1–5_), May (TEM_5_, PRE_5_), June to August (TEM_6–8_, PRE_6–8_), and August (TEM_8_, PRE_8_), were derived from the data of local weather stations.

## 3 RESULTS

### 3.1 Plant mass variations along the large‐scale gradient

**FIGURE 1:**
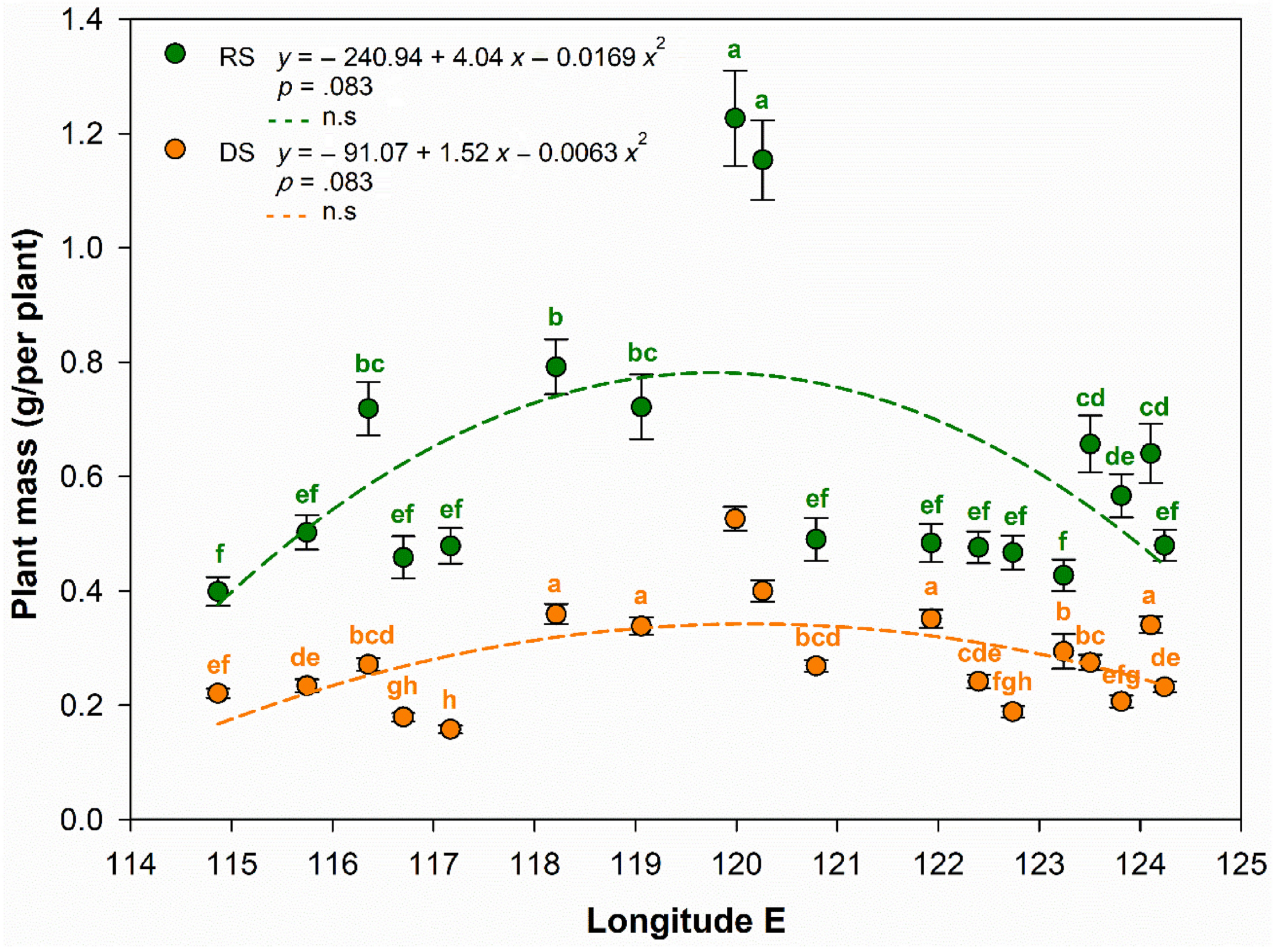
The variations of plant mass for *Leymus chinensis* along the longitude gradient, northeastern China. Bars with same letters in similar color indicate no significant differences between sites in dry season (DS)/in rainy season (RS).

PM in the species varied significantly along the large‐scale gradient (Figure 1). In general, PM increased first, peaking at site 11, and then dropped with the decrease of precipitation from east to west. Based on site‐by‐site analyses, PM showed significant differences among the sites except those of the adjacent ones (*p* < .05). PM at the east end (01 site, 0.23 g/per plant) was almost equal to that at the west end (18 site, 0.22 g/per plant, *F*_1,58_ = 0.88, *p* = .352) and was equivalent to only half of the middle (11 site, 0.53 g/per plant) in the DS (*F*_1,58_ = 165.60, *p* < .001). In the RS, PM at 01 site (0.48 g/per plant) was considerably greater than that at 18 site (0.40 g/per plant, *F*_1,58_ = 4.72, *p* = .034), while that at 11 site (1.23 g/per plant) was nearly threefold as those at sites from both ends. But those between adjacent sites (e.g., 2–4, 5–9, 14–18 sites) did not differ significantly (*p* > .05). PM from different grassland types also varied considerably at the gradient. The average PM at meadow sites (1–11 sites) was 0.30 g/per plant, while those at steppe (12–16) and desert (17–18) sites were 0.26 and 0.23 g/per plant in the DS. Similarly, the PM at meadow, steppe, and desert sites were 0.64, 0.63, and 0.45 g/per plant in the RS, respectively. There was no significant difference between steppe sites and desert sites in the DS (*F*_1,5_ = 0.25, *p* = .641), as well as between meadow sites and steppe sites in the RS (*F*_1,14_ = 0.004, *p* = .95).

The changing trends of PM were similar between the DS and RS along the gradient. PM for RS was significantly greater than that in the DS for each site (*p* < .01). PM at 01 and 18 sites were 52.1% and 45.0% greater in the RS, compared with those in the DS (*F*_1,58_ = 74.34, *p* < .001; *F*_1,58_ = 45.70, *p* < .001), and that at 11 site was as high as 56.9% (*F*_1,58_ = 65.89, *p* < .001) greater, respectively.

### 3.2 Climate effects on plant mass

**FIGURE 2:**
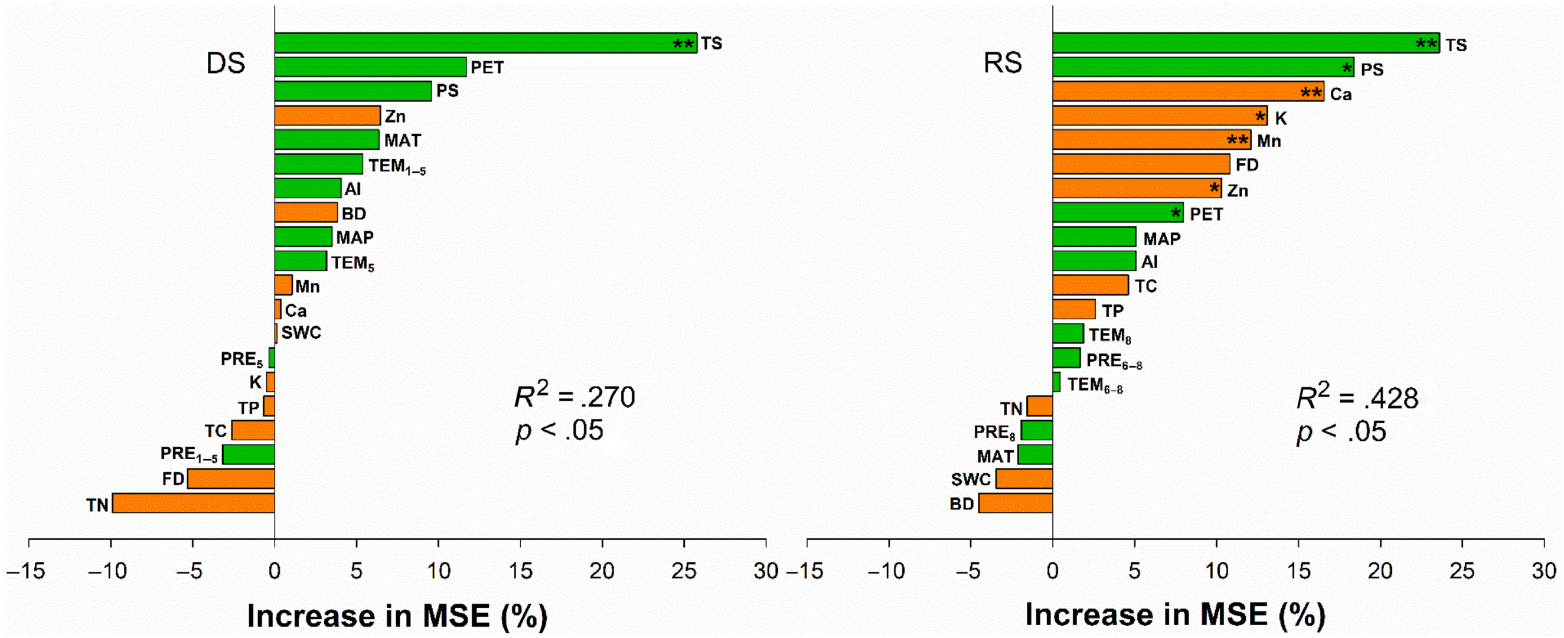
The relative importance of climatic variables, soil properties and microbial diversity for plant mass of *L. chinensis* in dry (DS) and rainy (RS) seasons, (*p < .05, **p < .01). AI, aridity index; BD, bacterial diversity; Ca, calcium contents; FD, fungi diversity; K, potassium content; MAP, mean annual precipitation; MAT, mean annual temperature; Mn, manganese content; PET, potential evapotranspiration; PRE_1–5_, all precipitations from January to May; PRE_5_, all precipitation in May; PRE_6–8_, all precipitations from June to August; PRE_8_, all precipitation in August; PS, precipitation seasonality; SWC, soil water content; TC, soil total C; TEM_1–5_, the average daily temperature from January to May; TEM_5_, the average daily temperature in May; TEM_6–8_, the average daily temperature from June to August; TEM_8_, the average daily temperature in August; TN, total N; TS, temperature seasonality; Zn, zinc contents.

**FIGURE 3:**
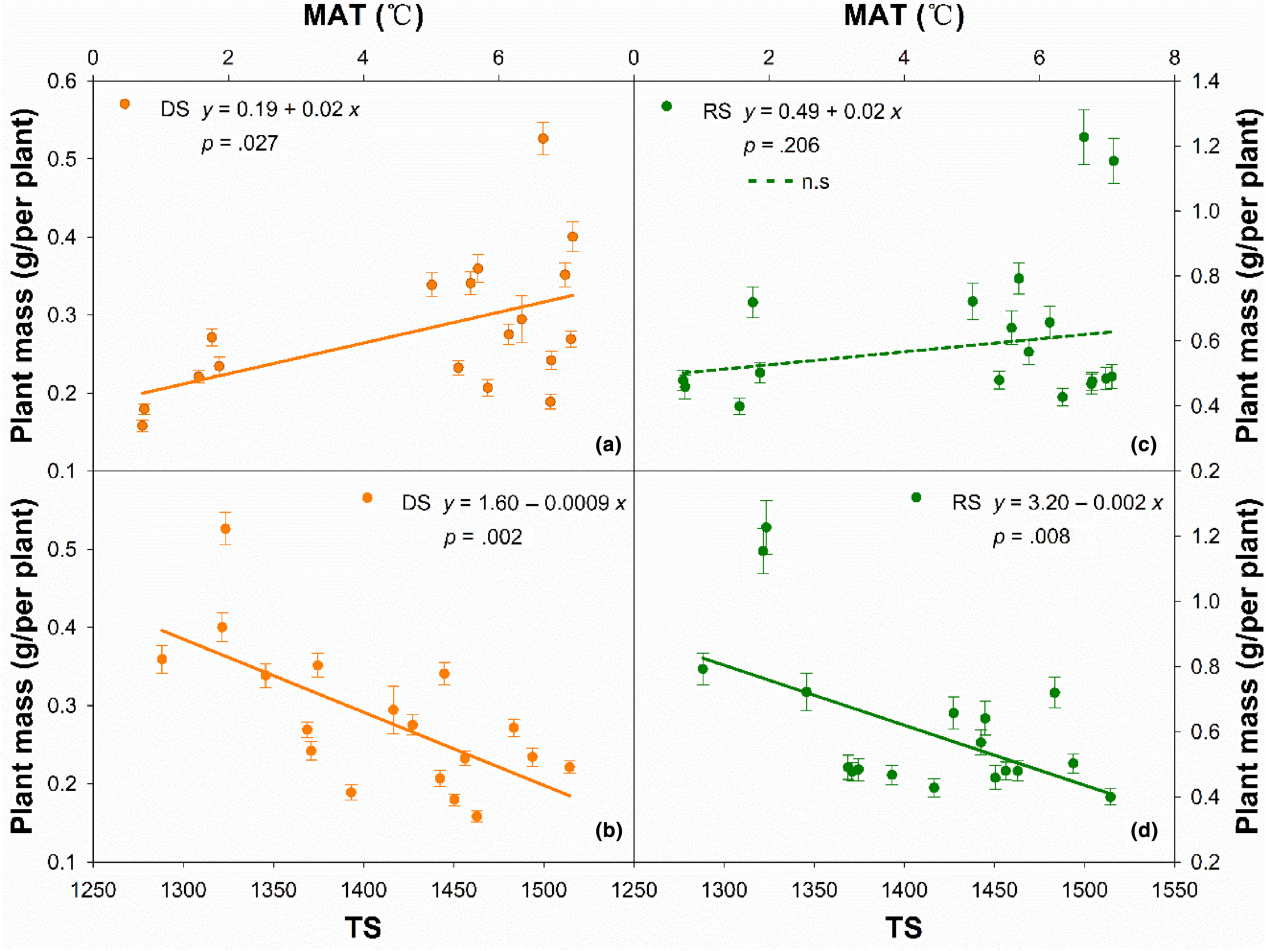
Relationships of plant mass for *L. chinensis* with mean annual temperature (MAT, a, c) and temperature seasonality (TS, b, d) in dry (DS) and rainy (RS) seasons along the large‐scale gradient.

Effects of climatic variables on PM were different in the DS and RS (Figure 2). Random forest analysis exhibited that temperature seasonality (TS) was the critical restriction of PM in the DS and RS (*p* < .01). Changing patterns predicted by PM of *L. chinensis* appear to be insensitive to some climate factors (e.g., MAT and PET) known to influence plant distribution in northeastern China (Table 2). The relations of PM against climate variables (e.g., temperature, precipitation, and aridity) by linear regression analyses were slightly different from those by random forest. In the DS, PM was strongly and positively correlated with MAT (*df* = 16, *r* ^2^ = .27, *p* = .027, Figure 3a), PS (*df* = 16, *r* ^2^ = .22, *p* = .047, Table 2) and PET (*df* = 16, *r* ^2^ = .40, *p* = .005, Table 2), but negatively and significantly with TS (*df* = 16, *r* ^2^ = .46, *p* = .002, Figure 3b). There were no significant relations between PM and precipitation parameters (MAP (*df* = 16, *r* ^2^ = .03, *p* = .506), PRE_1–5_ (*df* = 16, *r* ^2^ = .03, *p* = .467), and PRE_5_ (*df* = 16, *r* ^2^ = .04, *p* = .430)). In the RS, both MAT (*df* = 16, *r* ^2^ = .10, *p* = .206, Figure 3c and Table 2) and MAP (*df* = 16, *r* ^2^ < .001, *p* = .994, Figure 3c and Table 2) have much less impacts on PM, but PM was significantly and negatively correlated with TS (*df* = 16, *r* ^2^ = .36, *p* = .008, Figure 3d), strongly and positively with PS (*df* = 16, *r* ^2^ = .31, *p* = .017). Like those in the DS, there were no significant relations between PM and some climate variables (MAT (*df* = 16, *r* ^2^ = .10, *p* = .206), MAP (*df* = 16, *r* ^2^ < .001, *p* = .994), PET (*df* = 16, *r* ^2^ = .21, *p* = .056), TEM_6–8_ (*df* = 16, *r* ^2^ < .001, *p* = .924), PRE_6–8_ (*df* = 16, *r* ^2^ = .11, *p* = .178), and PRE_8_ (*df* = 16, *r* ^2^ = .07, *p* = .278)) in the RS.

**TABLE 2:** The relations of plant mass for *L. chinensis* with climate variables (*p < .05, **p < .01) in dry (DS) and rainy (RS) seasons along the large‐scale gradient.

Climate factors	PM in the DS	Climate factors	PM in the RS
Altitude	−0.2537	Altitude	−0.0325
MAT	0.5191*	MAT	0.3128
MAP	0.1678	MAP	0.0018
TS	−0.6754**	TS	−0.6003**
PS	0.4730*	PS	0.5559*
PET	0.6328**	PET	0.4586
AI	−0.0807	AI	−0.1715
TEM_1–5_	0.3566	TEM_6–8_	0.0242
TEM_5_	0.3484	TEM_8_	−0.0178
PRE_1–5_	−0.1830	PRE_6–8_	0.3322
PRE_5_	0.1983	PRE_8_	0.2701

Abbreviations: AI, aridity index; PET, potential evapotranspiration; PRE_1–5_, all precipitations from January to May; PRE_5_, all precipitation in May; PRE_6–8_, all precipitations from June to August; PRE_8_, all precipitation in August; PS, precipitation seasonality; TEM_1–5_, the average daily temperature from January to May; TEM_5_, the average daily temperature in May; TEM_6–8_, the average daily temperature from June to August; TEM_8_, the average daily temperature in August; TS, temperature seasonality (hereafter for figures).

### 3.3 Soil nutrient and microbial diversity impacts

**FIGURE 4:**
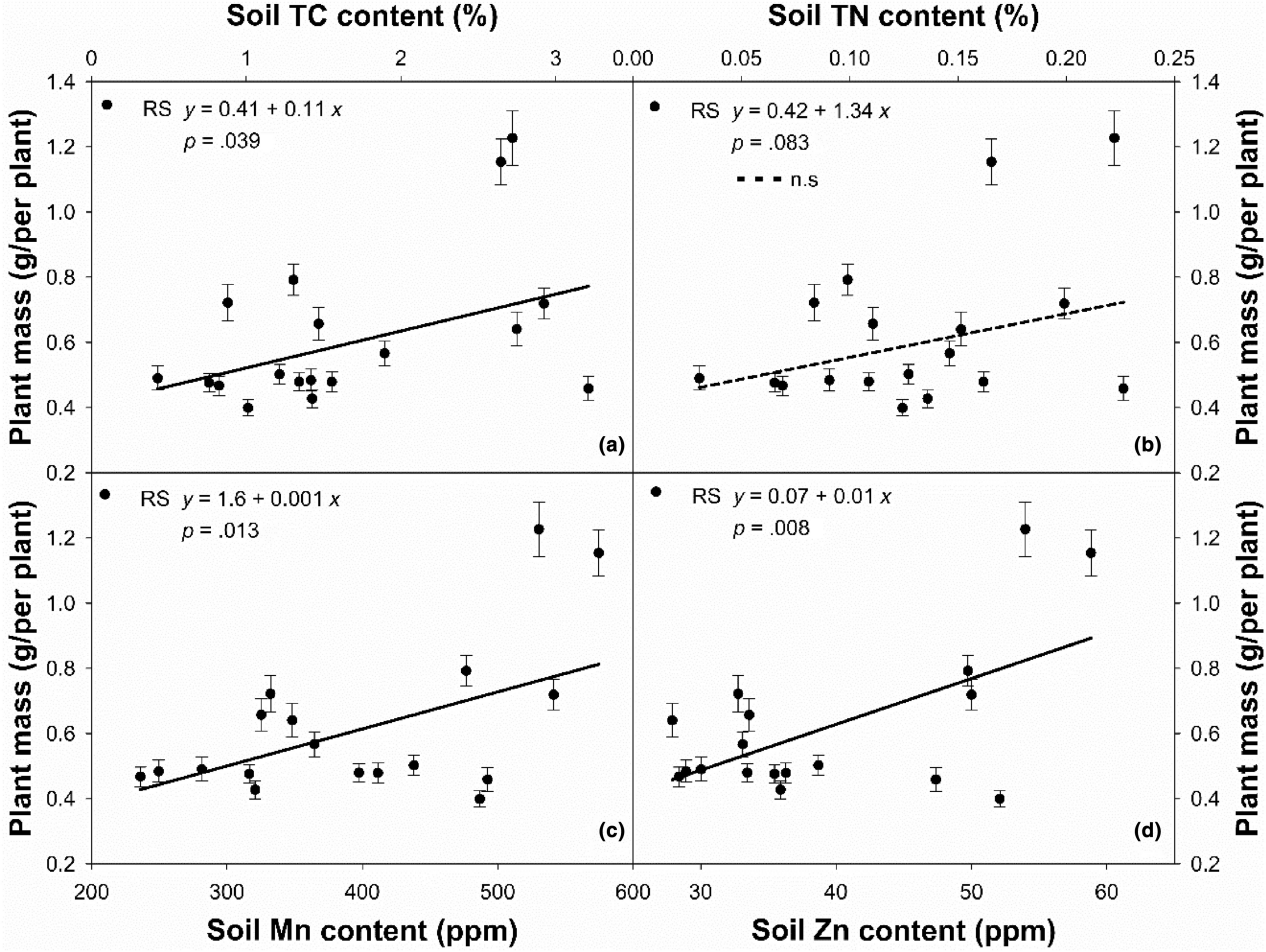
Correlations of plant mass for *L. chinensis* with soil total C (TC, a), total N (TN, b), Mn (c) and Zn (d) contents along the large‐scale gradient.

The effects of soil nutrients on PM of the species along the gradient were different in the DS and the RS (Table 3). There were no significant relations between PM and soil water content (SWC) at the gradient in both DS and RS. Soil resources (TN, TC, TP, Ca, K, Mn, Zn) also have much less impact on PM in the DS. On the contrary, soil TC (*df* = 16, *r* ^2^ = .24, *p* = .039, Figure 4a), Mn (*df* = 16, *r* ^2^ = .33, *p* = .013, Figure 4c), and Zn (*df* = 16, *r* ^2^ = .36, *p* = .008, Figure 4d) were positively and significantly correlated with PM of the species in the RS. Soil Ca and K contents were also significantly correlated with PM in the RS (*p* < .05), according to random forest analysis (Figure 2). In both DS and RS, there were no significant relations between PM and soil TN (*df* = 16, *r* ^2^ = .03, *p* = .469; *df* = 16, *r* ^2^ = .18, *p* = .082) and TP (*df* = 16, *r* ^2^ = .002, *p* = .864; *df* = 16, *r* ^2^ = .16, *p* = .101).

**TABLE 3:** The relationships of plant mass in *L. chinensis* with soil properties and microbial diversity (*p < .05, **p < .01) in dry (DS) and rainy (RS) seasons along the large‐scale gradient.

Soil properties in the DS	PM in the DS	Soil properties in the RS	PM in the RS
SWC	−0.1579	SWC	0.0850
TN	0.1825	TN	0.4203
TC	0.4121	TC	0.4904*
TP	−0.0436	TP	0.3989
Mn	0.0599	Mn	0.5743*
Zn	0.2463	Zn	0.6029**
Ca	0.3809	Ca	0.3660
K	−0.3509	K	−0.3880
BD	0.0851	BD	0.2325
FD	0.2240	FD	0.5240*

Abbreviations: BD, bacterial diversity; Ca, calcium; FD, fungi diversity; K, potassium; Mn, manganese; SWC, soil water content; TC, total carbon; TN, total nitrogen; TP, total phosphorus; Zn, zinc (hereafter for figures).

**FIGURE 5:**
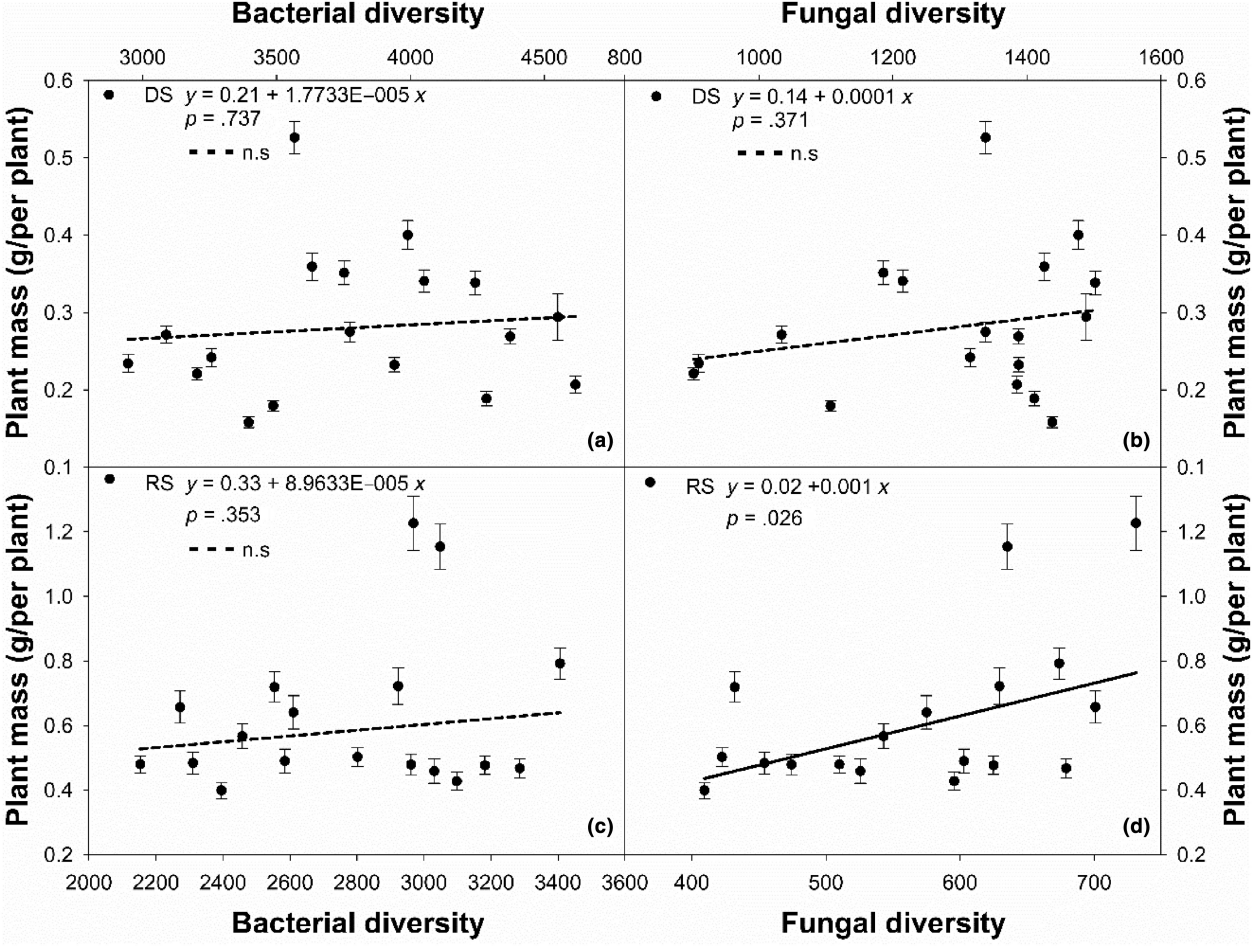
The correlations of plant mass for *L. chinensis* with soil bacteria diversity (a, c) and fungi diversity (b, d) along the large‐scale gradient.

Soil microbial diversity impacts on PM were slight in both DS and RS (Table 3). In the DS, PM was positively correlated with BD and FD, but not significant (*df* = 16, *r* ^2^ = .01, *p* = .737; *df* = 16, *r* ^2^ = .05, *p* = .371, Figure 5a,b). Similarly, there was no significant correlation between PM and BD in the RS, but PM was positively and significantly correlated with FD (*df* = 16, *r* ^2^ = .27, *p* = .026, Figure 5d).

## 4 DISCUSSION

Environmental changes, due to both natural and human activities, are known to affect plant distribution and establishment directly through influences on plant growth and reproduction (Callaway et al., 1994; Wang & Gao, 2003). Most studies pay attention to the effects of environmental variability on plant distribution, density, and community or species biomass, attempting to reveal changes in ecosystem structure and plant economic value in local scale (Callaway et al., 1994; Gao et al., 2020). However, only few addressed body size variations and its linkage with climate, soil nutrient, microbial diversity, combined with population traits in large‐scale gradient (Poulin, 1999; Schmid et al., 2000). This information is essential for our ability to evaluate the relative importance of environmental factors on bio‐distribution and predict climate change effects on species shift in the future.

Unlike the population densities which decreased linearly (Wang & Gao, 2003; Yuan et al., 2016), the changing trend of PM in *L. chinensis* exhibited a single peak curve shape in both DS and RS along the large‐scale gradient (Figure 1). This implies that the middle sites were the optimum distribution region for the species. Less PM variations between the western and the eastern sites suggested that the linear decrease of precipitation along the gradient was not the critical restriction regulating PM in the species. This and other studies (Wang & Gao, 2003; Yuan et al., 2016) clearly indicated that there was a trade‐off relation between PM and population densities of the species, which regulated plant survival and growth at steep moisture gradient. The adaptation strategy to drought of the species is by decreasing population densities and maintaining plant mass, especially in the arid western region (17 and 18 sites). The observed lower shoot densities in the dry sites are conducive to reducing water loss by plant transpiration (Wang & Gao, 2003), while maintaining PM at a certain level favors plants to synthetize more carbohydrates, thereby supporting shoot growth and tiller development in dry region, as well as plant distribution.

Similar changing patterns of PM for the species in both DS and RS along the gradient (Figure 1) also indicated that the PM variations were restricted to plant traits (e.g., population density, number of overwintering tiller) (Wang & Gao, 2003), not to precipitation. This finding was strengthened by the fact that there was no significant relationship between PM and precipitation (for both long term and short term) in both severe drought and humid rainy seasons. There is limited research on the interactive effects of precipitation and population density on plant mass for plants and additional experiments should be carried out in the future.

The results of random forest analysis not only confirmed the above explanation but also detected the divergences of driving factors of PM in the DS and RS (Figure 2). Previous research has shown that MAP was the main driving factor for vegetation and species distribution along a large‐scale gradient (Wang & Gao, 2003; Zhang et al., 1997). Our findings demonstrated that TS, in addition to PS and soil nutrients (Ca, K, Mn, Zn) in the RS, were the critical restriction of PM during growth season, suggesting that the driving mechanism of PM or plant growth was complex in natural grasslands (Stelling‐Wood et al., 2021). This result did not support our hypothesis that PM was confined to precipitation parameters at the gradient. This may explain why PM did not vary linearly as population densities with the decrease of MAP at the drought gradient in this study. Population densities may be reduced by density‐dependent or self‐thinning in the drier conditions when moisture is limited, for the tiller development of the species is strongly correlated with summer and fall precipitation (Wang et al., 2008; Wang & Gao, 2003). Because tiller production is the principal reproductive method in native grasslands, climate effects on shoot densities are mainly on the tiller numbers and tiller development, as well as tiller mortality (Wang & Gao, 2003). Previous research also suggested that climate mainly affected PM of *L. chinensis* indirectly by affecting plant traits (Yao et al., 2021).

Significant relationships of PM with TS in both DS and RS, as well as with PS in the RS, (Figure 3b,d) suggested that seasonal variation in temperature and precipitation were some of the main critical restrictions regulating PM of *L. chinensis* along the gradient. The studies implied that rain heat synchronization is conducive to plant growth in the dry region (Wang et al., 2008; Wang & Gao, 2003), assuming that the temperature and precipitation are within an appropriate range. Both high and low temperatures could cause significant losses in plant biomass and productivity (George et al., 2018), and strongly reduce cell size (Peter & Sommer, 2015). Both our findings and previous studies demonstrated that high TS was a hostile situation for plant growth (George et al., 2018). Although the drought caused by high temperatures may also limit plant growth, but the relative more precipitation at the same time definitely increases plant mass (Renaudin et al., 2022; Wang et al., 2008), this may explain why PM of the species was significantly and positively with PS along the gradient in the RS. In fact, the knowledge about the effects of temperature and precipitation seasonality on plant mass and growth is very limited and more experiments should be implemented.

Most soil properties have much less impact on PM in the DS and RS (Table 3), excepting that of TC, Mn, Zn, K and Ca contents in the RS, even though TN and TP were considered in short supply in *L. chinensis* grasslands (Wang et al., 2008). The effects of K and Ca contents on the species along the gradient have been well documented (Chen & Wang, 2009) and will not be elaborated here. Like population densities of the species, soil TN was linearly and positively correlated with MAP and clay content at the large‐scale gradient (Ma et al., 2014); this may partly explain why there was no significant correlation between PM and TN and TP at the gradient. Within an ecosystem independent of precipitation, the relationships between soil properties and plant biomass in natural grasslands are complex. It is consistently predicted that appreciable variation may not be due to one or even several soil properties in grassland biomass (Reinhart & Vermeire, 2017). On the contrary, soil TC, Mn, and Zn have significant effects on PM of the species in the RS (Figure 4). The amount of carbon sequestered in the soil increases in relation to plant biomass production (Cukor et al., 2022), as belowground particulate C addition significantly increases soil C mineralization rate and net N mineralization rate (Ma et al., 2014), which may favor of plant growth and increase PM. Both Mn and Zn are essential plant micronutrients and are involved in several physiological functions in plants (Tian et al., 2016). Mn is also considered a heavy metal that causes phytotoxicity when present in excess, disrupting photosynthesis and enzyme activity in plants. However, its toxicity was much less in grasses than in forbs, for Mn accumulation in forbs was about 10‐fold of that in grasses (Tian et al., 2016), due to differences in biochemical pathways regulating metal transport between dicots and monocots. Zn is one of the essential plant micronutrients and is involved in several physiological functions in plants, and the biochemical research demonstrated that Zn can increase plant growth by increasing photosynthesis and reducing oxidative stress (Rizwan et al., 2019). As of now, the information on trace element roles in natural grasslands is very limited. More physiological and biochemical experiments should be conducted to explore the effects of these trace elements on the species in the future.

It is not surprising that soil microbial diversity impacts on PM were slight in both DS and RS (Table 3), even though FD was positively and significantly correlated with PM (Figure 5d) in the RS. The previous research results showed that soil moisture and land‐use changes were most close related to microbial community composition and biomass from 23 sites at the large scale gradient, soil total C content and climate effects were weaker but still significant, while spatial structure, soil texture, nutrient availability, and vegetation types were not important (Ma et al., 2014). Our finding was partly supported by research that there was certain linkage between plant and soil fungal diversity along a large‐scale transect across grasslands, northern China (Wang et al., 2022). Our results suggest that soil properties and microbial diversity appear not to be the main restriction regulating plant mass of the species at the gradient.

## 5 CONCLUSION

The PM of *L. chinensis* varied in the shape of a single peak curve in both dry and rainy seasons along a large‐scale gradient, which was very different from population densities. Significant relationships between PM and TS and PS indicated that temperature and precipitation seasonality were the main limiting environmental factors, and not precipitation parameters (e.g., MAP, PRE_1–5_, PRE_5_, PRE_6–8_, and PRE_8_). These implied that the strategy of the species to adapt drought was by decreasing population densities and maintaining plant mass, especially in the arid western region. These findings imply that the distribution area of *L. chinensis* will not be reduced, that PM must be maintained at a certain level, and that population densities may significantly decrease if the drought in the region intensifies as predicted by the IPCC (2013).

## AUTHOR CONTRIBUTIONS

**Yuebin Zheng:** Formal analysis (equal); investigation (equal); writing – original draft (equal). **Jing Xue:** Investigation (equal). **Yixia Lv:** Investigation (equal). **Chaoxue Zhang:** Investigation (equal). **Renzhong Wang:** Conceptualization (lead); writing – original draft (lead); writing – review and editing (lead).

## FUNDING INFORMATION

This research was funded by grants from the National Natural Science Foundation of China (32071857).

## CONFLICT OF INTEREST STATEMENT

The authors declare that they have no conflict of interest.

## Supporting information


Data S1:

## Data Availability

Data's provided as supplementary material.

## References

[ece311215-bib-0001] Brown, R. P. , Znari, M. , El Mouden, E. , & Harris, P. (1999). Estimating asymptotic body size and testing geographic variation in Agama impalearis. Ecography, 22(3), 277–283.

[ece311215-bib-0002] Callaway, R. M. , Delucia, E. H. , & Schlesinger, W. H. (1994). Biomass allocation of montane and desert ponderosa pine – An analog for response to climate‐change. Ecology, 75(5), 1474–1481.

[ece311215-bib-0003] Cardou, F. , Munson, A. D. , Boisvert‐Marsh, L. , Anand, M. , Arsenault, A. , Bell, F. W. , Bergeron, Y. , Boulangeat, I. , Delagrange, S. , Fenton, N. J. , Gravel, D. , Hamel, B. , Hebert, F. , Johnstone, J. F. , Kumordzi, B. B. , Macdonald, S. E. , Mallik, A. , McIntosh, A. C. S. , McLaren, J. R. , … Aubin, I. (2022). Above‐ and belowground drivers of intraspecific trait variability across subcontinental gradients for five ubiquitous forest plants in North America. Journal of Ecology, 110(7), 1590–1605.

[ece311215-bib-0004] Chaves, M. M. , Maroco, J. P. , & Pereira, J. S. (2003). Understanding plant responses to drought ‐ from genes to the whole plant. Functional Plant Biology, 30(3), 239–264. 32689007 10.1071/FP02076

[ece311215-bib-0005] Chen, L. , & Wang, R. (2009). Anatomical and physiological divergences and compensatory effects in two *Leymus chinensis* (Poaceae) ecotypes in Northeast China. Agriculture, Ecosystems and Environment, 134, 45–52.

[ece311215-bib-0006] Cukor, J. , Vacek, Z. , Vacek, S. , Linda, R. , & Podrazsky, V. (2022). Biomass productivity, forest stability, carbon balance, and soil transformation of agricultural land afforestation: A case study of suitability of native tree species in the submontane zone in Czechia. Catena, 210, 105893.

[ece311215-bib-0007] Domros, M. , & Peng, G. B. (1988). The climate of China. Springer Verlag.

[ece311215-bib-0008] Fick, S. E. , & Hijmans, R. J. (2017). WorldClim 2: New 1‐km spatial resolution climate surfaces for global land areas. International Journal of Climatology, 37(12), 4302–4315.

[ece311215-bib-0009] Gao, X. L. , Liu, X. Q. , Ma, L. N. , & Wang, R. Z. (2020). Root vertical distributions of two Artemisia species and their relationships with soil resources in the Hunshandake desert, China. Ecology and Evolution, 10(6), 3112–3119. 32211181 10.1002/ece3.6135 PMC7083654

[ece311215-bib-0010] George, R. , Gullstrom, M. , Mangora, M. M. , Mtolera, M. S. P. , & Bjork, M. (2018). High midday temperature stress has stronger effects on biomass than on photosynthesis: A mesocosm experiment on four tropical seagrass species. Ecology and Evolution, 8(9), 4508–4517. 29760891 10.1002/ece3.3952 PMC5938440

[ece311215-bib-0011] Griffin‐Nolan, R. J. , Chieppa, J. , Knapp, A. K. , Nielsen, U. N. , & Tissue, D. T. (2023). Coordination of hydraulic and morphological traits across dominant grasses in eastern Australia. Functional Ecology, 37(4), 1126–1139.

[ece311215-bib-0012] Hounkpevi, A. , Salako, V. K. , Donhouede, J. C. F. , Dai, E. H. , Tovissode, F. , Kakai, R. G. , & Assogbadjo, A. E. (2020). Natural intraspecific trait variation patterns of the wild soursop *Annona senegalensis* (Annonaceae) along a climatic gradient in Benin, West Africa. Plant Ecology and Evolution, 153(3), 455–465.

[ece311215-bib-0013] IPCC . (2013). Climate change 2013: The physical science basis. Cambridge University Press.

[ece311215-bib-0014] Lande, R. (2009). Adaptation to an extraordinary environment by evolution of phenotypic plasticity and genetic assimilation. Journal of Evolutionary Biology, 22(7), 1435–1446. 19467134 10.1111/j.1420-9101.2009.01754.x

[ece311215-bib-0015] Liaw, A. , & Wiener, M. (2002). Classification and regression by random Forest. R News, 2, 18–22.

[ece311215-bib-0016] Ma, L. , Yuan, S. , Guo, C. , & Wang, R. (2014). Carbon and nitrogen dynamics of native *Leymus chinensis* grasslands along a 1000 km longitudinal precipitation gradient in northeastern China. Biogeosciences, 11(24), 7097–7106.

[ece311215-bib-0017] Peter, K. H. , & Sommer, U. (2015). Interactive effect of warming, nitrogen and phosphorus limitation on phytoplankton cell size. Ecology and Evolution, 5(5), 1011–1024. 25798219 10.1002/ece3.1241 PMC4364816

[ece311215-bib-0018] Poulin, R. (1999). Body size vs abundance among parasite species: Positive relationships? Ecography, 22(3), 246–250.

[ece311215-bib-0019] Reinhart, K. O. , & Vermeire, L. (2017). Power and limitation of soil properties as predictors of variation in peak plant biomass in a northern mixed‐grass prairie. Ecological Indicators, 80, 268–274.

[ece311215-bib-0020] Renaudin, M. , Blasi, C. , Bradley, R. L. , & Bellenger, J. P. (2022). New insights into the drivers of moss‐associated nitrogen fixation and cyanobacterial biomass in the eastern Canadian boreal forest. Journal of Ecology, 110(6), 1403–1418.

[ece311215-bib-0021] Rizwan, M. , Ali, S. , Rehman, M. Z. U. , & Maqbool, A. (2019). A critical review on the effects of zinc at toxic levels of cadmium in plants. Environmental Science and Pollution Research, 26(7), 6279–6289. 30635881 10.1007/s11356-019-04174-6

[ece311215-bib-0022] Schmid, P. E. , Tokeshi, M. , & Schmid‐Araya, J. M. (2000). Relation between population density and body size in stream communities. Science, 289(5484), 1557–1560. 10968792 10.1126/science.289.5484.1557

[ece311215-bib-0023] Stelling‐Wood, T. P. , Poore, A. G. B. , & Gribben, P. E. (2021). Shifts in biomass and structure of habitat‐formers across a latitudinal gradient. Ecology and Evolution, 11(13), 8831–8842. 34257931 10.1002/ece3.7714 PMC8258212

[ece311215-bib-0024] Tian, Q. Y. , Liu, N. N. , Bai, W. M. , Li, L. H. , Chen, J. Q. , Reich, P. B. , Yu, Q. , Guo, D. L. , Smith, M. D. , Knapp, A. K. , Cheng, W. X. , Lu, P. , Gao, Y. , Yang, A. , Wang, T. Z. , Li, X. , Wang, Z. W. , Ma, Y. B. , Han, X. G. , & Zhang, W. H. (2016). A novel soil manganese mechanism drives plant species loss with increased nitrogen deposition in a temperate steppe. Ecology, 97(1), 65–74. 27008776 10.1890/15-0917.1

[ece311215-bib-0025] Trabucco, A. , & Zomer, R. J. (2018). Global aridity index and potential evapotranspiration (ET0) climate database v2. CGIAR consortium for spatial information (CGIAR‐CSI) . Published online, available from the CGIAR‐CSI GeoPortal at https://cgiarcsi.community

[ece311215-bib-0026] Villalba, R. , & Veblen, T. T. (1998). Influences of large‐scale climatic variability on episodic tree mortality in northern Patagonia. Ecology, 79(8), 2624–2640.

[ece311215-bib-0027] Wang, C. W. , Ma, L. N. , Zuo, X. , Ye, X. H. , Wang, R. Z. , Huang, Z. Y. , Liu, G. F. , & Cornelissen, J. H. C. (2022). Plant diversity has stronger linkage with soil fungal diversity than with bacterial diversity across grasslands of northern China. Global Ecology and Biogeography, 31(5), 886–900.

[ece311215-bib-0028] Wang, R. Z. , Chen, L. , Bai, Y. G. , & Xiao, C. W. (2008). Seasonal dynamics in resource partitioning to growth and storage in response to drought in a perennial rhizomatous grass, *Leymus chinensis* . Journal of Plant Growth Regulation, 27(1), 39–48.

[ece311215-bib-0029] Wang, R. Z. , & Gao, Q. (2003). Climate‐driven changes in shoot density and shoot biomass in *Leymus chinensis* (Poaceae) on the north‐east China transect (NECT). Global Ecology and Biogeography, 12(3), 249–259.

[ece311215-bib-0030] Wang, R. Z. , & Ma, L. N. (2016). Climate‐driven C‐4 plant distributions in China: Divergence in C‐4 taxa. Scientific Reports, 6, 27977. 27302686 10.1038/srep27977 PMC4908390

[ece311215-bib-0031] Wang, R. Z. , & Ripley, E. A. (1997). Effects of grazing on a *Leymus chinensis* grassland on the Songnen plain of north‐eastern China. Journal of Arid Environments, 36(2), 307–318.

[ece311215-bib-0032] Yao, Z. Y. , Qing, H. , Yang, L. , & Zhao, L. Q. (2021). Non‐destructive aboveground biomass estimation of *Leymus chinensis* individual across large scale. Ecological Indicators, 131, 108212.

[ece311215-bib-0033] Yuan, S. , Guo, C. Y. , Ma, L. N. , & Wang, R. Z. (2016). Environmental conditions and genetic differentiation: What drives the divergence of coexisting *Leymus chinensis* ecotypes in a large‐scale longitudinal gradient? Journal of Plant Ecology, 9(5), 616–628.

[ece311215-bib-0034] Zhang, X. S. , Gao, Q. , Yang, D. A. , Zhou, G. S. , Ni, J. , & Wang, Q. (1997). A gradient analysis and prediction on the Northeast China transect (NECT) for global change study. Acta Botanica Sinica, 39, 785–799.

